# Comparative metabolomic and ionomic approach for abundant fishes in estuarine environments of Japan

**DOI:** 10.1038/srep07005

**Published:** 2014-11-12

**Authors:** Seiji Yoshida, Yasuhiro Date, Makiko Akama, Jun Kikuchi

**Affiliations:** 1 Graduate School of Medical Life Science, Yokohama City University, 1-7-29 Suehiro-cho, Tsurumi-ku, Yokohama, Kanagawa 230-0045, Japan; 2 RIKEN Center for Sustainable Resource Science, 1-7-22 Suehiro-cho, Tsurumi-ku, Yokohama, Kanagawa 230-0045, Japan; 3 RIKEN Biomass Engineering Program, 1-7-22 Suehiro-cho, Tsurumi-ku, Yokohama, Kanagawa 230-0045, Japan; 4 Graduate School of Bioagricultural Sciences, Nagoya University, 1 Furo-cho, Chikusa-ku, Nagoya, Aichi 464-0810, Japan

## Abstract

Environmental metabolomics or ionomics is widely used to characterize the effects of environmental stressors on the health of aquatic organisms. However, most studies have focused on liver and muscle tissues of fish, and little is known about how the other organs are affected by environmental perturbations and effects such as metal pollutants or eutrophication. We examined the metabolic and mineral profiles of three kinds of abundant fishes in estuarine ecosystem, yellowfin goby, urohaze-goby, and juvenile Japanese seabass sampled from Tsurumi River estuary, Japan. Multivariate analyses, including nuclear magnetic resonance-based metabolomics and inductively coupled plasma optical emission spectrometry-based ionomics approaches, revealed that the profiles were clustered according to differences among body tissues rather than differences in body size, sex, and species. The metabolic and mineral profiles of the muscle and fin tissues, respectively, suggest that these tissues are most appropriate for evaluating environmental perturbations. Such analyses will be highly useful in evaluating the environmental variation and diversity in aquatic ecosystems.

Estuarine ecosystems provide important services (e.g., aquatic productivity, wildlife habitat, food supply) that cannot be replicated artificially. Estuaries are considered to be of high value because of the functions they provide^1^,^2^,^3^, but they exhibit low long-term resilience. Thus, natural restoration and recovery processes are often too slow to support the demand for resources following habitat degradation and biodiversity loss resulting from construction, fishery collapse, and disasters such as the 2011 Japanese Tsunami. As a result, managers often undertake artificial restoration (with large associated costs)^2^. In this context, there is an increasing need for methods of evaluating the status of estuarine environments to allow timely implementation of conservation actions and sustainable management of these ecosystems.

Environmental metabolomics (or metabonomics) provides a method for characterizing the interactions between organisms and their environments^4^,^5^,^6^. This approach offers a number of advantages for studying organism–environment interactions and in assessing metabolic function and homeostasis at the molecular fingerprinting level^4^. In particular, nuclear magnetic resonance (NMR)-based metabolomics approaches, which can be incorporated into environmental monitoring and chemical risk assessments, can generate high-quality data for use in environmental regulatory evaluations^7^,^8^. The benefits of NMR-based metabolomics approaches include observation of high-abundance metabolites that contain nonexchangeable hydrogen atoms, measurement of potentially quantitative metabolites with a high degree of reproducibility, relatively high throughput and automated analyses, and the existence of established technologies with minimal instrument downtime^9^. NMR-based metabolomics approaches also yield spectra that are, in principle, comparable between laboratories throughout the world. This is because the chemical shift measured by NMR is a physical quantity that can be measured with good reproducibility. These benefits suggest that an NMR-based approach has significant utility for environmental studies^7^. In a comparison of metabolomics analytical techniques, researchers detected 108 unique metabolites using an NMR-based approach, and the other 88 and 28 metabolites were overlapped with the metabolites detected by GC-MS and DFI/LC-MS/MS, respectively^10^. This highlights the differing ability of NMR- and MS-based approaches for detecting certain metabolites. NMR-based metabolomics approaches have been used both in environmental studies and to evaluate homeostasis in humans and other animals^11^,^12^,^13^,^14^,^15^,^16^. Assessment of estuarine and aquatic ecosystems using an NMR-based metabolomic approach will be likely to aid in determining the environmental effects of pharmaceuticals and other chemicals on aquatic organisms. Further, the approach has already increased our understanding of fish physiology, development, disease, and responses to water pollution^17^,^18^,^19^,^20^,^21^,^22^,^23^,^24^,^25^,^26^.

Depending on their concentrations, various minerals are either essential or potentially toxic to estuarine organisms; homeostatic mechanisms are required to regulate the intracellular levels of these substances. For example, cells require nutrients (e.g., phosphorus, sulfur, and selenium) as components of macromolecules, as cofactors required for enzymatic activity (e.g., copper and iron), for neurotransmitter function (e.g., calcium), and for structural integrity (e.g., zinc). Thus, investigation of mineral profiles is important to the evaluation of estuarine ecosystems. Recently, a number of study fields (such as plant science and microbiology) have incorporated an assessment of the mineral profiles by ionomic, mineral, or trace element analysis^27^,^28^,^29^,^30^,^31^,^32^. It is important to measure both inorganic elements and organic metabolites because these reflect both the condition of the physical environment and that of the organisms living in the environment. Here, we applied ionomic analyses in combination with an NMR-based metabolomics approach to evaluate estuarine ecosystems.

Environmental metabolomic or ionomic approaches have been used to examine changes in aquatic organisms in response to exposure to heavy metals in laboratory studies^33^,^34^ and natural environments^35^, and to assess accumulation of heavy metals and trace elements in liver and muscle tissues of wild and hatchery fish^36^,^37^,^38^,^39^,^40^,^41^. However, little is known regarding the potential of other fish tissues to assess environmental conditions and responses. To address this challenge, we have chosen three kinds of abundant fishes in estuarine ecosystem, yellowfin goby (*Acanthogobius flavimanus*), urohaze-goby (*Glossogobius olivaceus*), and juvenile Japanese seabass (*Lateolabrax japonicus*) as test organisms for environmental monitoring in the Tsurumi River estuary, Japan. Yellowfin and urohaze-goby are primary consumers in this ecosystem and are appropriate for use in our experiment because these fish are demersal. We hypothesized that the metabolic profiles of these species should strongly reflect environmental conditions.

Our objective was to measure the metabolic and mineral profiles in various tissues and compare the profiles among the three species. Japanese seabass are more migratory than the gobies and we thus expected that the species profiles would differ. We compared the metabolic and mineral profiles of each tissue and fish species using inductively coupled plasma optical emission spectrometry (ICP-OES) and NMR with multivariate analyses (Fig. 1). The tissues that were most appropriate and practical for metabolic and mineral profiling were selected for obtaining metabolotypes that reflected concentrated environmental information.

## Results

### Mineral profiles

**Figure 2:**
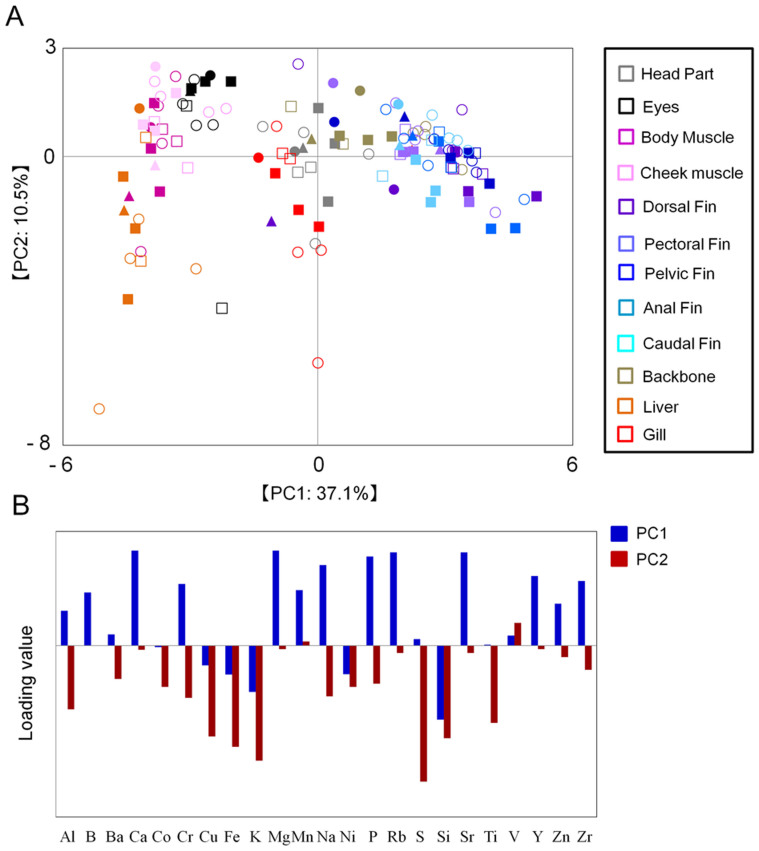
Mineral profiles of fish tissues based on ICP-OES analytical data. The principal components analysis (PCA) score plot (A) and loading plot (B) of the three fish species are displayed. A total of 132 samples were used for analysis. Circles, urohaze-goby; triangles, yellowfin goby; squares, Japanese seabass; closed symbols, female; open symbols, male; gray, head parts; black, eyes; purple, body muscle; pink, cheek muscle; blues (light to dark), each fin; red, gill; orange, liver; ocher, backbone.

The mineral profiles of yellowfin goby, urohaze-goby, and juvenile Japanese seabass were measured using ICP-OES-based ionomic analysis of head, eye, gill, cheek muscle, body muscle, backbone, liver, dorsal fin, pectoral fin, pelvic fin, anal fin, and caudal fin tissues (Figs. S1–S3). The Principal component analysis (PCA) score plot of ICP-OES data demonstrated that the mineral profiles were likely to cluster according to differences among body tissues rather than differences in sex, body size (body mass index), or species (Fig. 2A). The mineral profiles of all of the fin types clustered in a PC1-positive direction, while those of cheek and body muscles, eye, and liver clustered in a PC1-negative direction. The mineral profile of the fin tissues was more diverse than those of other tissues. The loading plot analysis revealed that many minerals (e.g., Mg, Na, and P) were more abundant in fin tissue than in other tissues (Fig. 2B), particularly in comparison with muscles (Table 1). These data suggest that fin tissue has the most value for assessing environmental conditions.

Next, we evaluated interspecific variation in the mineral profile of the body muscle and fin tissues. The muscle profiles clustered by species, without overlap (Fig. S4A). There were significant differences in the body muscle profiles between species for a number of minerals, including Al, Cr, Cu, Mg, Mn, S, and Zn (Figure S4B). Because the variation in the profiles was similar among the different fin tissues in the PCA, the data for different fin types were combined for this evaluation. The mineral profiles in the PCA plot of ICP-OES fin data clustered clearly by species (Fig. S4C). The differences between species were significant for a number of minerals, including Ba, Ca, Cr, Cu, Fe, K, Mn, Na, P, S, and Sr (Fig. S4D). Taken together, our observations suggest that the mineral profiles of the body muscle and fin tissues captured the interspecific variation among the three fish species lived in the Tsurumi River estuary.

### Metabolic profiles

**Figure 3:**
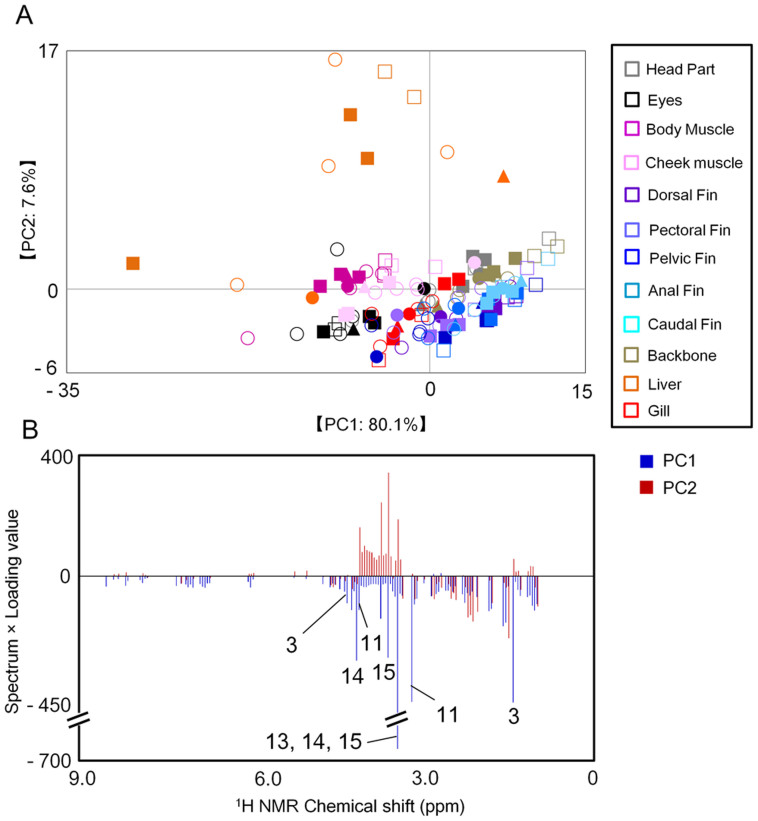
Metabolic profiles of fish tissues based on ^1^H-NMR spectra. The principal components analysis (PCA) score plot (A) and loading plot (B) of the three species are displayed. A total of 132 samples were used for analysis. Numbers on loading plots represent the metabolites listed in Table 2. Circles, urohaze-goby; triangles, yellowfin goby; squares, Japanese seabass; closed symbols, female; open symbols, male; gray, head parts; black, eyes; purple, body muscle; pink, cheek muscle; blue (light to dark), each fin; red, gill; orange, liver; ocher, backbone.

The metabolic profiles of the three fish species were evaluated using NMR-based metabolomic analysis of head, eye, gill, cheek muscle, body muscle, backbone, liver, dorsal fin, pectoral fin, pelvic fin, anal fin, and caudal fin tissues. The NMR spectral data were digitized and statistically computed for PCA; the PCA score plot demonstrated that the metabolic profiles were also likely to cluster according to differences among body tissues rather than differences in sex, body size (body mass index), or species (Fig. 3A). The metabolic profiles of head, gill, backbone, and fins clustered in a PC1-positive and PC2-negative direction; the eye, cheek, and body muscle profiles clustered in a PC1-negative direction and liver profiles clustered in a PC1-negative and PC2-positive direction. The profiles of the eye, cheek and body muscles, and liver were more diverse than those of the other tissues. The factors contributing to separation in the PCA scores were also analyzed by loading plots (Fig. 3B). To confirm the metabolic annotation for body muscle, we conducted an ^1^H–^13^C HSQC NMR experiment using juvenile Japanese seabass (Fig. S6); the annotated metabolites are provided in Table S1. The metabolic profiles of the eye, cheek and body muscles, and liver included lactate, creatine, taurine, trimethylamine N-oxide (TMAO), and betaine (Table 2), indicating that these tissues contained more information on genetic and environmental differences among individuals than did other tissues. Metabolic profiling of eye, cheek and body muscle, and liver can thus provide valuable information on environmental conditions. Because most fish have a higher volume of body muscle tissue than eye, cheek muscle, or liver tissue, body muscle is better suited for metabolic profiling by NMR to evaluate environmental metabolotypes (particularly for small fish).

Next, we evaluated interspecific variation of the metabolic profiles in body muscle. The NMR spectra of the WG pulse sequences encompassed a broad range, from approximately 0.5 to 2.5 ppm, possibly-derived from proteins and peptides (Fig. S5). To better observe small molecules, CPMG spectra were acquired to attenuate broad signals from macromolecules and to enhance the sharper signals (Fig. S5). The PCA score plot of NMR spectra of the WG and CPMG pulse sequences showed clear clustering of metabolic profiles by species (Fig. S7A, C). The differences between juvenile Japanese seabass and the two gobies were greater than those between the two goby species in the PCA plots. We observed significantly higher levels of TMAO (3.25 ppm) and lower levels of alanine (1.45 ppm) and glycine (3.53 ppm) in the muscles of the juvenile Japanese seabass compared with those of the gobies (Fig. S7B, D).

**Figure 4:**
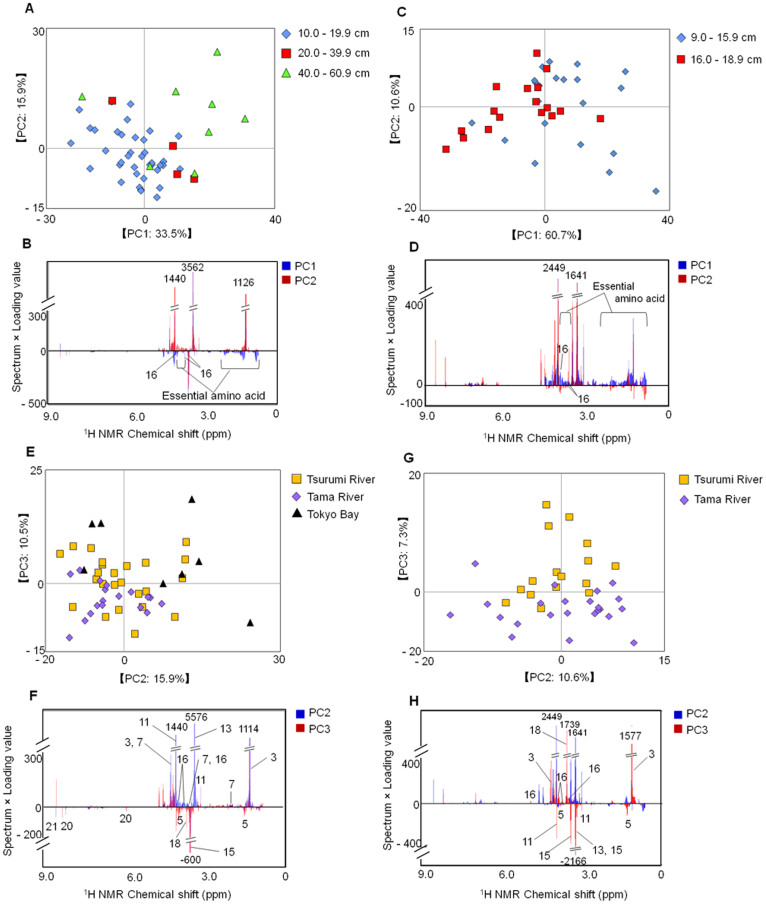
Metabolic profiles of body size of Japanese seabass (n = 50) and yellowfin goby (n = 38) based on ^1^H-NMR spectra. The PCA score plots (A and C) and loading plots (B and D) of the two fish species are displayed. Numbers on loading plots represent the metabolites listed in Table 2 (A,B): Japanese seabass; diamonds, 10.0–19.9 cm; squares, 20.0–39.9 cm; triangles, 40.0–60.9 cm; (C,D): yellowfin goby; diamonds, 9.0–15.9 cm; squares, 16.0–18.9 cm. (E–H): Metabolic profiles of Japanese seabass (n = 50) and yellowfin goby (n = 38) from different collection locations based on NMR spectra. The PCA score plots (E and G) and loading plots (F and H) of the two species are displayed. Numbers on loading plots indicate the annotated metabolites listed in Table 2. (E, F:) Japanese seabass; squares, Tsurumi River; diamonds, Tama River; triangles, Tokyo Bay; (G, H): yellowfin goby; squares, Tsurumi River; diamonds, Tama River.

Furthermore, we evaluated intraspecific differences in the metabolic profiles of yellowfin goby and Japanese seabass. The PCA revealed metabolic clustering according to body size (growth stage) (Fig. 4A, C). Yellowfin gobies < 16.0 cm and Japanese seabass < 40.0 cm were contributed by some metabolites such as essential amino acids and d-glucose (Fig. 4B, D). PCA plots of intraspecific variation revealed that Japanese seabass clustered according to habitat, although there was some overlap among fish from the Tama and Tsurumi river estuaries (Fig. 4E). Japanese seabass collected from the Tsurumi River estuary and Tokyo Bay were characterized by lactate, TMAO, d-glucose, proline, and creatine, whereas the same species in the Tama River estuary were characterized by alanine, taurine, glycine, IMP, and AMP (Fig. 4F). The metabolic profiles of yellowfin goby were likely to be clustered based on the differences between Tsurumi and Tama River estuaries (Fig. 4G and Fig. S8A). From the loading plot analyses, the metabolites such as lactate, glycine, alanine, creatine, TMAO, and taurine contributed to the profiles (Fig. 4H and Fig. S8B). These metabolites may be affected by environmental factors such as nutrient concentrations, salt levels, pH, river flow rate, and population density.

In addition to measuring water-soluble metabolites, we also conducted a metabolomic analysis of non-polar metabolites in the body muscle of yellowfin goby using methanol extraction followed by ^1^H-NMR measurements (Fig. S9). The metabolic profiles were separated into two groups based on the location of collection (Tama and Tsurumi river estuaries) (Fig. S9A). The loading plot analysis revealed that the levels of phospholipids (such as phosphatidylcholine) and fatty acids (such as linoleate) were high in yellowfin goby from the Tsurumi River estuary (Fig. S9B and Table S2). Furthermore, partial least squares (PLS) regression model was constructed based on the metabolomic profiles of the water- and methanol-soluble metabolites to validate the profiling in two river ecosystems (Table S3). The PLS models constructed using training sets were validated by 10-fold cross validation. The validation using external datasets indicated that accuracy rate using test sets was 90.91% for both water- and methanol-metabolomic profiles on the PLS regression models. Thus, it was suggested that the profilings targeting for water- and methanol-soluble metabolites enabled us to discriminate the different ecosystems on Tsurumi and Tama Rivers from environmental metabolotypes.

## Discussion

We measured the metabolic and mineral profiles in a range of fish tissue to describe the environmental metabolotypes. Our results indicated that the mineral profiles of fin tissue and the metabolic profiles of body muscle and fin tissues offered the most utility for environmental evaluation. In addition, using the NMR-based metabolomic approach to measure both water-soluble polar metabolites and methanol-soluble non-polar metabolites, we successfully characterized the environmental metabolotypes from two different estuary ecosystems. The use of advanced environmental assessment technologies such as those presented here will enable capture of environmental data and optimization of aquatic ecosystem services.

We observed higher levels of TMAO and lower levels of alanine and glycine in muscles of juvenile Japanese seabass compared with those of gobies based on a comparison of body muscle profiles between species (Fig. S7). TMAO is a well-known osmolyte in marine species^42^, and amino acids, including alanine and glycine, are related to gluconeogenesis and energy metabolism^43^. Our observations suggested that differences in metabolites likely represent behavioral differences among species (i.e., juvenile Japanese seabass exhibit greater migratory activity than gobies).

Some metabolites (e.g., essential amino acids and d-glucose) were abundant in yellowfin goby < 16.0 cm and in Japanese seabass < 40.0 cm. This result suggested that these metabolites might be important for, or at least be related to, growth during juvenile stages.

The metabolic and mineral profiles of the body muscle and fin tissues, respectively, were most appropriate for evaluation of environmental dynamics. Each tissue has a different turnover rate because of tissue-specific rates of cell synthesis and catabolism, as shown by stable isotope experiments using ^13^C or ^15^N. For example, splanchnic tissues have faster turnover rates than structural tissues^44^,^45^,^46^. Because the turnover rates of organic compounds and muscle metabolites are lower in the muscle than in fins, plasma, and liver^45^, we speculate that muscle turnover rates provide a better representation of the environment. In contrast, many elements have a long biological half-life relative to organic compounds. For example, the biological half-life of Cd is 10–30 years^47^, suggesting that the mineral profiles obtained from fin tissue is reflective of relatively long-term exposure to environmental factors compared with the metabolic profiles. Furthermore, in contrast to fin mineral profiles, the metabolic profile of the body muscles may better reflect information about short-term exposure to environmental variables, such as cumulative dietary information or biological or chemical stressors.

The metabolic and mineral profiling described here provides an important tool for environmental assessment, and is an example of integrated NMR-based analysis combined with an ICP-OES-based approach for mining environmental information. This approach enables evaluation of environmental variation and diversity in aquatic ecosystems. In future studies, we intend to characterize environmental variation among estuarine ecosystems using this method with test organisms such as gobies. These studies will examine relationships between fish and environmental parameters such as metabolites and nutrients.

## Methods

### Sample collection and processing

Fish were collected from September to December in 2011 and 2012 from the Tsurumi River estuary, Yokohama City, Kanagawa, Japan (35°29′52.8″ to 35°30′14.4″N, 139°40′37.2″ to 139°40′44.4″E), and the nearby Tama River estuary, Kawasaki City, Kanagawa, Japan (35°32′27.6″ to 35°32′45.6″N, 139°44′16.8″ to 139°45′07.2″E). We also collected Japanese seabass (40.0–60.9 cm) in January 2014 in Tokyo Bay (35°26′49.2″N, 139°49′51.6″E), between the Tsurumi and Tama rivers. Following collection, each fish was identified as a yellowfin goby, urohaze-goby, or juvenile Japanese seabass. The species inhabiting only the Tsurumi River were processed, dissected, and separated into head parts (excluding eye, gill, and cheek muscle), eye, gill, cheek muscle, body muscle, backbone, liver, dorsal fin, pectoral fin, pelvic fin, anal fin, and caudal fin (Figures S1–S3). Only body muscle was analyzed in terms of yellowfin goby and Japanese seabass collected from the Tama River and Tokyo Bay. Each tissue was lyophilized and crushed for 10–30 min to a powder using an Automill (Tokken Inc., Chiba, Japan). Liver samples were washed with hexane and evaporated using a centrifugal evaporator (Eyela, Tokyo Rikakikai Co. Ltd., Japan) because lipids that are abundant in the liver inhibit lyophilization.

### ICP-OES measurement

Residues of samples extracted for NMR measurements were incubated with 6 mL of aqueous nitric acid (6.9% v/v) in a Thermomixer comfort (Eppendorf Japan, Tokyo, Japan), as described previously^48^. The collected supernatants were filtered through a Millex GS filter (0.22 μm, EMD Millipore, Billerica, MA) for ICP-OES analysis, which was performed using a SPS5510 (Hitachi High-Tech Science Corporation, Tokyo, Japan) as described prevously^49^.

### NMR measurement

To characterize water-soluble metabolites, 5 (for tissue profiling) or 30 (for comparisons of estuarine environments) mg of each powdered sample was extracted with 700 μL of PBS (0.1 M K_2_HPO_4_/KH_2_PO_4_, pH 7.0) containing 90% deuterium oxide with 1 mM sodium 2,2-dimethyl-2-silapentane-5-sulfonate (DSS) internal standard at 65°C for 15 min. To characterize methanol-soluble metabolites, 30 mg of each powdered sample was extracted with 1000 μL of deuterated methanol (99.8%, Cambridge Isotope Laboratories Inc., MA, USA) with 1 mM DSS internal standard at 55°C for 15 min. After centrifugation at 25°C for 5 min, the extracted supernatant was transferred to a 5-mm NMR tube. One-dimensional (1D) Watergate (WG) and Carr–Purcell–Meiboom–Gill (CPMG) spectra were acquired at 298 K using a Bruker AVANCE II 700 spectrometer equipped with a ^1^H inverse triple-resonance cryogenically cooled probe with Z-axis gradients (Bruker BioSpin GmbH, Rheinstetten, Germany), as described previously^50^. In brief, 32 K data points with a spectral width of 9803 Hz were collected into 32 transients and 8 dummy scans. Prior to Fourier transformation, free induction decays were multiplied by an exponential window function corresponding to a 0.3-Hz line-broadening factor. The acquired spectra were manually phased and baseline corrected. The method for NMR measurement of two-dimensional ^1^H-^13^C heteronuclear single quantum coherence (HSQC) has been described previously^51^,^52^. In brief, a total of 512 complex F1 (^13^C) and 1024 complex F2 (^1^H) points were recorded from 128 scans per increment. The F1 and F2 spectral widths were 150 and 14 ppm, respectively. NMR spectra were processed using Bruker TopSpin software (Bruker BioSpin GmbH) and assigned using the SpinAssign program at the PRIMe website (http://prime.psc.riken.jp/)^53^,^54^,^55^ and the Biological Magnetic Resonance Bank (http://www.bmrb.wisc.edu/metabolomics/query_metab.php)^56^.

### Statistical analysis

All 1D ^1^H-NMR data were reduced by subdividing the spectra into sequential 0.04- or 0.01-ppm designated regions between ^1^H chemical shifts of 0–9.5 ppm. After exclusion of water resonance, each spectrum was normalized by an internal standard or constant sum^57^. PCA were performed on the R platform (R Development Core Team, 2012) as described previously^58^,^59^,^60^. The data were visualized as PCA score plots and loading plots. Each coordinate on the score plots represented an individual sample, and each coordinate on the loading plots represented one NMR spectral data point related to the metabolites or identified minerals in the ICP-OES measurements. Analyses of PLS regression models were performed on the R with package “pls”. The *Y*-matrix was generated by the value of one (when the sample *i* belongs to the Tsurumi River) and zero (when the other elements of the row *i*) matrix. The parameters for the PLS models were provided in Table S3.

## Author Contributions

S.Y., Y.D. and J.K. designed research; S.Y. and Y.D. performed experiments; S.Y. and M.A. analyzed data and made the figures; S.Y., Y.D. and J.K. wrote the paper. All authors reviewed the manuscript and agreed with the submission.

## Supplementary Material

Supplementary Information Supporting information

## Figures and Tables

**Figure 1:**
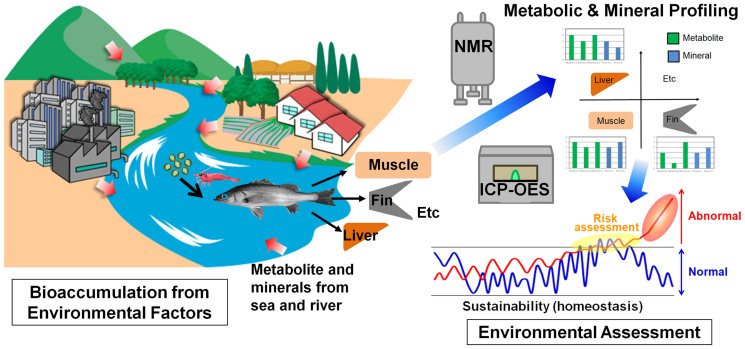
Conceptual diagram illustrating environmental assessment from metabolic and mineral perturbations of wild fish tissues using ^1^H-nuclear magnetic resonance (NMR) and inductively coupled plasma optical emission spectrometry (ICP-OES) techniques with multivariate analysis. The metabolic and mineral perturbations in wild fishes can be occurred from river waters as well as sea tide through bioaccumulation of natural food web system. Comparative analysis of these perturbations can assess range of normal and risk of environmental threats. (Drawings by Seiji Yoshida, Yasuhiro Date and Jun Kikuchi).

**Table 1:** Major minerals observed in fish muscle and fins

		*Gobiidae*				*Percichthyidae*				
		Muscle (ppm)		Fin (ppm)		Muscle (ppm)		Fin (ppm)		
No.	Element	Value	SE*	Value	SE*	Value	SE*	Value	SE*	Elemental function
1	Al	10.13 ± 3.88		22.65 ± 6.43		4.96 ± 1.40		15.84 ± 3.60		
2	Ba	1.41 ± 0.89		3.02 ± 0.11		1.20 ± 0.64		2.97 ± 0.07		
3	Ca	1029.72 ± 317.87		50467.02 ± 1973.39		2308.91 ± 1097.15		67315.44 ± 1662.71		Constitutive element
4	Cr	0.48 ± 0.07		1.10 ± 0.07		0.71 ± 0.06		1.32 ± 0.04		Enzymatic element
5	Cu	2.97 ± 1.81		2.10 ± 0.30		1.36 ± 0.11		2.08 ± 0.45		Enzymatic element
6	Fe	12.50 ± 6.02		24.90 ± 6.45		6.71 ± 0.92		10.27 ± 0.64		Enzymatic element
7	K	24556.99 ± 3766.49		14922.84 ± 446.63		26461.21 ± 2363.95		17321.60 ± 538.17		Electrolytic element
8	Mg	82.93 ± 6.95		1395.22 ± 58.39		108.98 ± 19.81		1494.96 ± 37.18		Electrolytic element
9	Mn	0.95 ± 0.16		19.04 ± 0.99		0.51 ± 0.07		2.99 ± 0.09		Enzymatic element
10	Na	262.95 ± 40.88		739.94 ± 31.07		346.05 ± 42.97		1153.16 ± 49.17		Electrolytic element
11	P	13420.09 ± 1945.85		42126.89 ± 1534.37		14579.14 ± 1434.68		54545.62 ± 1281.87		Constitutive element
12	S	377.93 ± 41.03		370.42 ± 20.38		465.63 ± 36.85		458.35 ± 12.93		Constitutive element
13	Si	207.13 ± 19.89		99.22 ± 5.37		211.84 ± 10.97		93.60 ± 3.04		Constitutive element
14	Sr	4.85 ± 1.58		341.51 ± 14.14		11.85 ± 6.00		518.82 ± 15.08		
15	V	0.10 ± 0.03		1.28 ± 0.16		0.05 ± 0.04		1.11 ± 0.08		Enzymatic element
16	Zn	14.55 ± 0.69		104.31 ± 9.35		20.44 ± 1.92		137.51 ± 16.48		Enzymatic element

*SE: standard error.

**Table 2:** Annotated metabolites exhibiting significantly high and low PCA loading values in Fig. 2B

No.	Compound	^1^H chemical shift (ppm)	multiplicity	Spectrum × Loading value of PC1	Spectrum × Loading value of PC2
1	Valine	1.04	d	−66.76	34.11
		2.29	m	−34.77	−82.54
		3.62	d	−28.31	55.45
2	Leucine	0.96	d	−118.27	−39.40
		1.69	m	−112.22	−93.25
		3.7	t	−28.65	80.38
3	Lactate	1.33	d	−432.96	57.93
		4.1	q	−93.11	3.70
4	Lysine	1.45	m	−159.68	−39.37
		2.97	t	−97.46	−79.28
		3.78	t	−37.68	88.37
5	Alanine	1.45	d	−159.68	−39.37
		3.78	q	−37.68	88.37
6	Arginine	1.69	m	−112.22	−93.25
		3.21	t	−60.69	56.57
7	Proline	2.01	m	−53.19	−156.43
		3.37	t	−27.18	67.58
		3.41	t	−279.75	354.08
		4.14	t	−53.34	−17.59
8	Glutamate	2.05	m	−67.18	−133.90
		2.13	m	−91.65	−38.39
		2.37	dt	−36.18	−77.99
9	Glutamine	2.17	m	−62.29	1.97
		2.45	m	−50.47	−0.67
		3.78	m	−37.68	88.37
10	Malate	2.33	dd	−54.42	−58.46
		4.3	dd	−29.54	−30.00
11	Creatine	3.01	s	−430.68	7.96
		3.9	s	−116.62	166.33
12	Choline	3.21	s	−60.69	56.57
		3.49	t	−28.50	70.66
13	Trimethylamine N-Oxide (TMAO)	3.25	s	−632.16	194.25
14	Betaine	3.25	s	−632.16	194.25
		3.89	s	−51.89	−20.71
15	Taurine	3.25	t	−632.16	194.25
		3.45	t	−25.10	76.97
16	D-Glucose	3.41	t	−279.75	354.08
		3.53	dd	−146.44	252.20
		3.74	dd	−31.89	83.46
		3.82	m	−37.71	103.28
		5.22	d	−9.66	18.08
17	Myo-inositol	3.53	dd	−146.44	252.20
		3.62	dd	−28.31	55.45
		4.02	t	−116.60	−44.52
18	Glycine	3.53	s	−146.44	252.20
19	Serine	3.86	dd	−34.11	81.81
		3.98	m	−51.89	−20.71
20	IMP	4.02	m	−116.60	−44.52
		4.38	m	−38.54	−30.91
		4.5	m	−28.08	−2.87
		6.15	d	−39.33	7.63
		8.23	s	−33.20	12.26
		8.36	s	−10.38	8.37
21	AMP	4.02	m	−116.60	−44.52
		4.38	m	−38.54	−30.91
		4.50	m	−28.08	−2.87
		8.56	s	−36.32	0.48
