# Supplementary material for: Comparative metabolomic and ionomic approach for abundant fishes in estuarine environments of Japan
Source: Sci Rep. 2014 Nov 12;4:7005. doi: 10.1038/srep07005 (PMC4228347; doi:10.1038/srep07005)
Supplement: Supplementary Information — Supporting information [file srep07005-s1.doc]

**Supporting Information**

**Comparative metabolomic and ionomic approach for abundant fishes in estuarine environments of Japan**

**Seiji Yoshida^†^, Yasuhiro Date^†, ‡^, Makiko Akama^‡^, and Jun Kikuchi^†, ‡, §, ║^, ***

^†^Graduate School of Medical Life Science, Yokohama City University, 1-7-29 Suehiro-cho, Tsurumi-ku, Yokohama, Kanagawa 230-0045, Japan

^‡^RIKEN Center for Sustainable Resource Science, 1-7-22 Suehiro-cho, Tsurumi-ku, Yokohama, Kanagawa 230-0045, Japan

^§^RIKEN Biomass Engineering Program, 1-7-22 Suehiro-cho, Tsurumi-ku, Yokohama, Kanagawa 230-0045, Japan

^║^Graduate School of Bioagricultural Sciences, Nagoya University, 1 Furo-cho, Chikusa-ku, Nagoya, Aichi 464-0810, Japan

*Corresponding author, Jun Kikuchi: RIKEN Center for Sustainable Resource Science, 1-7-22 Suehiro-cho, Tsurumi-ku, Yokohama, Kanagawa 230-0045, Japan. Tel: +81455039439; Fax: +81455039489; E-mail: [jun.kikuchi@riken.jp](mailto:jun.kikuchi@riken.jp)

**Figure S1.** Anatomy of *Lateolabrax japonicus*. Photograph was taken by Seiji Yoshida.

**Figure S2.** Anatomy of *Acanthogobius flavimanus.* Photograph was taken by Seiji Yoshida.

**Figure S3.** Anatomy of *Glossogobius olivaceus.* Photograph was taken by Seiji Yoshida.

**Figure S4.** Mineral profiles in the body muscle (A, B) and fin (C, D) tissues based on inductively coupled plasma optical emission spectrometry (ICP-OES) analytical data. The principal component analysis (PCA) score plots (A, C) and relative intensities of each mineral (B, D) are displayed. A total of 15 samples were used for analysis. Differences between two groups (*: P<0.05, **: P<0.01) were assessed using Student’s t-test. Squares, yellowfin goby; circles, urohaze-goby; triangles, Japanese seabass.

**Figure S5.** Representative ^1^H NMR spectra of the body muscles of Japanese seabass (A, B); urohaze-goby (C, D); and yellowfin goby (E, F) measured using Carr–Purcell–Meiboom–Gill (CPMG; A, C, E) and Watergate (WG; B, D, F) pulse-sequence programs. Numbers on the spectra represent the annotated metabolites (listed in Table 2).

**Figure S6.** Representative ^1^H-^13^C heteronuclear single quantum coherence (HSQC) NMR spectra of the body muscle of *Lateolabrax japonicus*.

**Figure S7.** Metabolic profiles of the body muscles based on ^1^H-NMR spectra using WG (A, B) and CPMG (C, D) pulse sequence programs. The PCA score plots (A, C) and relative intensities of each metabolite (B, D) are displayed. A total of 15 samples were used for analysis. Differences between two groups (*: P<0.05, **: P<0.01) were assessed using Student’s t-test. Squares, yellowfin goby; circles, urohaze-goby; triangles, Japanese seabass.

**A**

**B**

**Figure S8.** ^1^H-NMR spectra-derived metabolic profile of the water-soluble components in the body muscle of yellowfin goby (n=170) captured in the Tama river or the Tsurumi river. The PCA score plot (A) and loading plot (B) are displayed. The numbers on the loading plots represent the annotated metabolites listed in Table 2.

**A**

**B**

**Figure S9.** ^1^H-NMR spectra-derived metabolic profile of the methanol-soluble components in the body muscle of yellowfin goby (n=176) captured in the Tama river or the Tsurumi river. The PCA score plot (A) and loading plot (B) are displayed. The numbers on the loading plots represent the annotated metabolites listed in Table S2.

**Table S1.** Annotated water-soluble metabolites detected in the HSQC-NMR spectra

**Table S2.** Annotated methanol-soluble metabolites detected in the HSQC-NMR spectra

**Table S3.** Parameters and validation results of PLS models

|  | Number of samples for training sets | | k | A | R^2^ | Q^2^ | Number of samples for test sets | | Accuracy rate using external validation (%) |
| --- | --- | --- | --- | --- | --- | --- | --- | --- | --- |
|  |  |  |  |  |  |  |  |  |  |
|  | Tsurumi | Tama |  |  |  |  | Tsurumi | Tama |  |
| Water | 61 | 65 | 872 | 19 | 0.97 | 0.88 | 22 | 22 | 90.91 |
| Methanol | 66 | 66 | 911 | 24 | 0.97 | 0.85 | 22 | 22 | 90.91 |
